# Access to and self-reported health impacts of COVID-19 prevention measures on people with disabilities in Vietnam: Results from a cross-sectional study in three mega-cities

**DOI:** 10.1186/s12889-026-27167-w

**Published:** 2026-04-14

**Authors:** Sarah Marks, Vu Quynh Mai, Luong Anh Ngoc, Xanthe Hunt, Divya Goyal, Hoang Van Minh, Lena Morgon Banks

**Affiliations:** 1 International Centre for Evidence in Disability (ICED), London School of Hygiene and Tropical Medicine, London, UK; 2 Hanoi University of Public Health, Hanoi, Vietnam; 3 The Fred Hollows Foundation, Sydney, Australia; 4 Center for Training and Research on Substance Abuse - HIV, Hanoi Medical University, Hanoi, Vietnam; 5 Centre for Global Mental Health, London School of Hygiene and Tropical Medicine, London, UK; 6 Department of Global Health, Institute for Life Course Health Research, Stellenbosch University, Stellenbosch, South Africa; 7 New Delhi, India

**Keywords:** Disability inclusion, COVID-19 impacts, Vietnam, Healthcare access, Morbidity

## Abstract

**Background:**

COVID-19 and measures for its containment have had direct and indirect impacts on people’s health, with certain groups disproportionately impacted. This cross-sectional study in Vietnam assessed self-reported health impacts of COVID-19 related restrictions amongst people with and without disabilities, and their inclusion in response measures.

**Methods:**

A cross-sectional survey conducted from December 2021 to January 2022 included 898 participants from Da Nang, Ha Noi, and Ho Chi Minh City in Vietnam. Disability status was determined using government disability cards, the Washington Group short set questions, or self-identification. Analysis compared self-reported health impacts (e.g., well-being, access to general and disability related health services) and inclusion in response measures (e.g., access to vaccination, personal protective equipment) between people with and without disabilities using StataSE 17.

**Results:**

People with disabilities were much more likely to report negative impacts of COVID-19 restrictions on their well-being (Prevalence Ratio (PR) 1.8, 95% CI 1.5–2.3), and general healthcare access (PR 2.6, 95% CI 1.8–3.8). Despite similar vaccination rates, people with disabilities faced challenges in vaccine information access (PR 4.4, 95% CI 2.5–7.8), appointment scheduling, site accessibility and interactions with staff (PR 11.9, 95% CI 4.8–29.5). They also encountered difficulties adhering to preventive measures, although they were more likely to receive personal protective equipment and hygiene products compared to people without disabilities (PR 2.9, 95% CI 2.0-4.1).

**Conclusions:**

People with disabilities reported significantly worse well-being and healthcare access and faced greater barriers in accessing vaccination information and services despite similar vaccination coverage. These findings indicate that while high vaccination coverage is achievable, disproportionate structural barriers faced by people with disabilities call for inclusive, targeted strategies in future public health emergencies.

**Supplementary Information:**

The online version contains supplementary material available at 10.1186/s12889-026-27167-w.

## Background

Globally, the COVID-19 pandemic and measures for its containment have had profound impacts on the health and well-being of people with disabilities [1–3]. People with disabilities faced heightened risks of negative health consequences during periods of heightened restrictions. For example, they were more affected by disruptions to routine healthcare, including rehabilitation and impairment-related services, as they are more likely to require these services [4–6]. Other barriers to accessing health services also increased, such as reduced social support resulting from COVID-19 control measures [5, 6]. Moreover, the economic impact of COVID-19 related restrictions disproportionately affected people with disabilities, many of whom were already more likely to be living in poverty pre-pandemic [7, 8]. Consequently, people with disabilities experienced higher morbidity and mortality rates than the general population [1–3], being 2.7 (95% CI 2.4–3.2) times more likely to die from COVID-19 [3].

Vietnam’s response to the COVID-19 pandemic demonstrated early preparedness and decisive action. It was one of the first countries to declare a national epidemic and implement early restrictions to reduce transmission, such as border control, contact tracing, quarantines, mask mandates, and medical declarations [9–14]. Between January 2020 and March 2021, Vietnam experienced minimal cases and deaths, particularly during the first three waves, despite sharing a land border with China, thanks to these early strict measures [9, 14, 15]. Despite early success in controlling COVID-19, subsequent outbreaks created considerable challenges [11, 12, 14]. The fourth wave, which spanned from April 2021 to March 2022, resulted in over 10 million cases and 42,454 deaths, revealing the limits of the initial containment efforts [15].

While the Vietnamese government was initially effective in curbing transmission, these measures profoundly impacted lives of people in Vietnam and in particular people with disabilities [6, 8, 16]. The aim of this study was to explore the self-perceived impact of COVID-19 and related restrictions on the health of people with disabilities in comparison to people without disabilities in three mega-cities of Vietnam and their experiences following control measures such as vaccination, social distancing and masking.

It was hypothesised that the impact of COVID-19 on health would be perceived to be worse amongst people with disabilities than those without disabilities due to existing inequities in access to health services for people with disabilities in Vietnam and insufficient inclusion in response strategies [17, 18]. By capturing and comparing the experiences and perceptions of people with disabilities, we seek to provide valuable insights into the heightened health challenges they encountered during the pandemic. It is hoped that these insights will inform both national and global health strategies, ensuring that the needs of people with disabilities are not overlooked in emergency response planning and public health initiatives in the future.

## Methods

### Study design

A cross-sectional telephone survey was conducted to determine the social and economic impact of the COVID-19 pandemic amongst people with and without disabilities (see Additional File 1 for questionnaire). The primary outcome of interest for the study was the self-perceived impact of the COVID-19 pandemic on access to healthcare among people with disabilities in comparison to people without disabilities. Secondary outcomes of interest were self-perceived impact of COVID-19 on health, access to COVID-19 prevention measures, education, employment, household finances and access to social protection. This analysis focuses on self-perceived inclusion in COVID-19 prevention measures and the impact of COVID-19 on health, with economic impacts reported elsewhere [8].

The study used a quota-based sampling method with the aim of recruiting 500 people with disabilities matched with 500 people without disabilities (1000 participants in total) from the three largest cities of Vietnam: Da Nang, Ha Noi and Ho Chi Minh City, with participants approximately equally divided across the three cities. Based on this sample size, the study had 80% power at a 95% confidence level to detect a minimum difference of approximately 9% points in self-perceived inclusion in COVID-19 prevention measures between people with and without disabilities, assuming a conservative overall estimate of 50% access to COVID-19 prevention measures.

### Participant recruitment

Participants were recruited through quota-based sampling. People with disabilities were initially identified through Organisations of Persons with Disabilities (OPDs) in each city. Participants were purposively selected to maximise heterogeneity based on gender, location, age, and impairment type (sensory, physical, cognitive and psychosocial). However, as mobility impairments predominated amongst OPD members, additional recruitment was conducted through non-governmental organisations focused on other impairment types and ages e.g. blindness, deafness, and children with disabilities. People with disabilities were matched to people without disabilities by gender, approximate age, and location using frequency matching. Participants without disabilities were identified through general population lists obtained from the provincial health departments in each city.

All participants were contacted by phone and invited to participate remotely due to restrictions on in-person meetings at the time of data collection. All individuals were then screened for disability using the Washington Group Short Set on Functioning questions over the phone [19], as well as a further question on whether they considered themselves to have a disability and/or had a disability identification card. People with disabilities were defined as anyone having any of the following: a government-issued disability card; screened positive for disability using the Washington Group Short Set questions, with the recommended cut-off of “a lot of difficulty” in at least one functional domain of seeing, hearing, walking, remembering, communicating, or self-care [19]; or self-reported they had a disability. People who did not meet any of these criteria were defined as not having a disability. If a person recruited from the general population responded positively to one of the aforementioned criteria, they were reclassified as a person with disability.

Oral recorded consent was provided by all participants (or their caregivers) prior to survey commencement. Caregivers consented and/or responded as a proxy on behalf of people with certain impairments who had significant difficulties with communication and comprehension. Sign language interpretation via video call was available for individuals using sign language, who had access to the required technology. Respondents were provided with mobile phone data to engage in the call. The questionnaire explored the self-reported impact of the COVID-19 pandemic on people with and without disabilities across each domain of interest.

## Outcome measures

The following indicators were assessed:

- Self-reported impact on health and access to healthcare: participants were asked how COVID-19 measures impacted their (1) well-being and (2) access to needed services, including services linked to their disability (medications, health services, assistive products, personal assistance) compared to pre-pandemic. Responses used a 5-step Likert scale on reported impact of COVID-19 with options ranging from “a lot better” to “a lot worse”. Binary variables were created of “a lot worse” vs. no change/better/a little worse.
- Access to vaccination: participants report being fully vaccinated (i.e. receiving full recommended dosage; participants receiving less than recommended dosage classified as not being vaccinated).
- Challenges accessing vaccination: participants were asked if they had difficulty with (1) accessing information about vaccines; (2) getting an appointment for vaccination; (3) traveling to the vaccination site; and (4) interactions with staff at site.
- Challenges with preventive measures: participants were asked about their level of difficulty with (1) masking; (2) social distancing; and (3) handwashing. Options were “no”, “some” and “a lot of difficulty”, and binary variables of “any difficulty” vs. “no difficulty” were generated.
- Receipt of personal protective equipment: participants were asked if they had received personal protective equipment (e.g., masks, hygiene kits) from the government or any organisation.
- Symptoms, testing and diagnosis of COVID-19: participants were asked if they had experienced symptoms of COVID-19 (fever, sore throat), and if so, if they had taken a test for COVID-19. Participants who took a test were asked for the result (positive or negative for COVID-19).

### Data collection

Data collection was conducted between 15th December 2021 and 30th January 2022, during the fourth wave of the COVID-19 pandemic [14], by 10 trained interviewers from Ha Noi University of Public Health. A total of 1761 people were invited to participate, of which 898 (51%) agreed to participate in the study. Data was collected from 479 people with disabilities and 419 people without disabilities. Each interview lasted about 15–20 min, sometimes longer if interviews required sign-language interpretation, with data captured electronically by the data collectors using REDCap.

### Data analysis

Data was analysed using StataSE17. Data was disaggregated by disability status and prevalence ratios (PR) comparing outcomes between people with and without disabilities were generated using a modified Poisson regression approach with robust error variance [20]. Controls for age group, gender and location (Ha Noi, Da Nang or Ho Chi Minh City) were included in the regression. Analyses on indicators related to vaccination were restricted to adults (aged 18 years of older) only given eligibility for vaccinations.

## Results

The demographic characteristics of the participants with disabilities was similar to those without disabilities in terms of the distribution across locations and gender (Table 1). However, participants with disabilities were generally older and poorer, likely reflecting the higher prevalence of disability among older age groups and the economic disadvantages faced by people with disabilities [21–23]. Amongst those with disabilities, approximately 86% had a disability card – of which the majority had a severe or extremely severe designation of disability on their card and just over half had a physical impairment.

**Table 1:** Demographic characteristics of participants by disability status (*N* = 898)

	Disability (*n* = 479)	No Disability (*n* = 419)
Gender		
Male	47.6%	43.0%
Female	52.4%	57.0%
Age group		
0–17	12.3%	13.1%
18–39	27.4%	51.1%
40–64	48.2%	29.4%
65+	12.1%	6.4%
Location		
Ha Noi	39.8%	36.5%
Ho Chi Minh City	30.1%	33.4%
Da Nang	30.1%	30.1%
Household poverty designation		
Non-poor	74.5%	95.7%
Near poor	9.8%	2.6%
Poor	15.7%	1.7%
Disability card details		
Has a disability card	86.2%	-
Disability card level		
Mild	15.0%	-
Severe	57.6%	-
Extremely severe	18.6%	-
Unknown	8.7%	-
Disability type		
Physical	54.7%	-
Hearing	10.7%	-
Vision	17.4%	-
Psychological	7.5%	-
Intellectual	8.5%	-
Other	7.1%	-

People with disabilities were 1.8 times more likely (95% CI 1.5–2.3) to report that COVID-19 impacted their well-being and 2.6 times more likely (95% CI 1.8–3.8) to report that COVID-19 impacted their access to general medicines and healthcare (Table 2). Amongst people with disabilities who required healthcare related to their disability, 35.1% reported having significantly worse access to health services, 21.2% reported significantly worse access to personal assistance, 19.4% to medications, and 14.4% to assistive products compared to pre-pandemic.

**Table 2:** Participants that self-reported the impact of COVID-19 restriction measures on their overall health and healthcare access as “significantly worse”

	Disability (*n* = 479)	No Disability (*n* = 419)	PR (95% CI)
Well-being	37.2%	19.1%	1.8 (1.5–2.3)
Access to general medicines and healthcare	22.8%	7.9%	2.6 (1.8–3.8)
Access to disability-related health services^a^			
Medications	19.4%	-	-
Health services	35.1%	-	-
Assistive products	14.4%	-	-
Personal assistance	21.2%	-	-

^a^Amongst people who reported needing it

People with disabilities were more likely to report experiencing difficulties with getting COVID-19 vaccine information (PR 4.4, 95% CI 2.5–7.8), obtaining a vaccination appointment (PR 1.8, 95% CI 1.1-3.0), traveling to the vaccination appointment (PR 24.5, 95% CI 9.2–65.7), waiting at the vaccination site (PR 2.4, 95% CI 1.7–3.5), and interacting with staff at the vaccine site (PR 11.9, 95% CI 4.8–29.5) (Table 3).

Regarding other COVID-19 preventive measures, people with disabilities were more likely to experience difficulties with adherence including for wearing masks (PR 1.6, 95% CI 1.2–2.1), social distancing (PR 1.5, 95% CI 1.2–1.7) and following handwashing and hygiene protocols (PR 3.6, 95% CI 2.1–6.2).

**Table 3:** Participants that self-reported “any difficulty” following COVID-19 preventive measures

	Disability	No Disability	PR (95% CI)
Access to vaccines (Adults only)	*n* = 420	*n* = 364	
Getting information	16.4%	3.9%	4.4 (2.5–7.8)
Getting an appointment	11.4%	6.6%	1.8 (1.1-3.0)
Traveling to appointment site	28.1%	1.1%	24.5 (9.2–65.7)
Waiting at vaccine site	19.8%	8.8%	2.4 (1.7–3.5)
Interactions with staff at vaccination centre	13.1%	1.4%	11.9 (4.8–29.5)
Following other preventive measures (All ages)	*n* = 479	*n* = 419	
Wearing a face mask	21.3%	14.8%	1.6 (1.2–2.1)
Regular handwashing/hygiene protocols	12.9%	3.6%	3.6 (2.1–6.2)
Social distancing	46.0%	30.1%	1.5 (1.2–1.7)

Despite reported difficulties in accessing vaccines, people with disabilities were equally likely (PR 1.0, 95% CI 0.9–1.1) to report that they were fully vaccinated compared to people without disability and 1.6 times more likely (95% CI 1.2–2.1) to have received personal protective equipment (PPE) or hygiene products. People with disabilities were less likely to self-report having symptoms of COVID-19 compared to people without disabilities and were equally likely to report being tested but less likely to be diagnosed with COVID-19 (Table 4). Among participants who reported being tested for COVID-19, for those with disabilities 77.0% reported using rapid tests and 11.7% polymerase chain reaction (PCR) tests, whereas for people without disabilities 58.3% reported using rapid tests and 41.7% PCR tests.

**Table 4:** Self-reported participant experiences of COVID-19 prevention, symptoms and diagnosis

	Disability (*n* = 479)	No Disability (*n* = 419)	Pr (95% CI)
Received PPE or hygiene products^a^	24.6%	16.5%	1.6 (1.2–2.1)
Fully vaccinated (Adults only)	89.2%	88.5%	1.0 (0.9–1.1)
Symptoms of COVID-19	14.8%	23.4%	0.7 (0.5–0.9)
Tested for COVID-19 (*if had symptoms*)	87.3%	84.7%	1.0 (0.9–1.1)
Diagnosed with COVID-19	8.8%	17.9%	0.5 (0.4–0.7)

^a^Received from government or any other organisation

## Discussion

One of the key factors behind Vietnam’s early success was strong government leadership and the rapid mobilization of resources [10]. The implementation of a national COVID-19 Response Plan allowed for timely testing, tracing, and quarantine programs, supported by effective public health communication through mobile applications and media campaigns [9]. The country’s prior investments in public health, particularly following the Severe Acute Respiratory Syndrome (SARS) epidemic, played a crucial role in maintaining public trust and ensuring compliance with preventive measures [9]. However, implementing strict containment measures for COVID-19 carried other challenges for maintaining health and access to health services that disproportionately affected people with disabilities – which this study reveals. People with disabilities also faced additional challenges adhering to preventive measures and navigating the vaccination process - although promisingly vaccination rates were high amongst both people with and without disabilities.

### Impact of COVID-19 on healthcare access for people with disabilities

The strict lockdowns and social distancing measures in Vietnam severely disrupted healthcare services, particularly services considered less critical like rehabilitation and routine health screenings [10]. To mitigate these disruptions, telemedicine and home-based care were implemented, however home visits were implemented late [24]. Despite this, people with disabilities were found in this study to be disproportionately affected by these healthcare disruptions, being 2.6 times more likely (95% CI 1.8–3.8) to report facing difficulties obtaining needed healthcare. Research from other settings has also found that COVID-19 related disruptions to health systems particularly affected people with disabilities, who often have greater healthcare needs [6]. Some of these challenges are health system related, as services were re-oriented towards COVID-19 related prevention and care, while others could be due to factors such as social distancing mandates, fear of visiting health facilities due to infection risk, and loss of personal assistance to seek needed products and services. Other strategies implemented for COVID-19 prevention may have not been inclusive of people with disabilities. An example of this was the use of contact-tracing apps for entering public places [25]– such as health facilities. Although not a prerequisite for services, as alternative manual tracing was offered where needed, the use of contact-tracing apps overlooked the fact that not everyone could afford internet access due to depleted incomes during the pandemic [25]. This would have disproportionately affected people with disabilities [8, 26, 27]. Furthermore, many features of these apps were inaccessible to screen-reader or talk-back users [25]. These barriers highlight the urgent need for integrating disability rights into pandemic preparedness and recovery plans to ensure equitable access to healthcare and information for people with disabilities.

### Access and adherence to COVID-19 prevention and treatment interventions

A key strength of Vietnam’s response was its communication regarding COVID-19, leveraging traditional media and mobile applications to reach its citizens [10]. SMS alerts and the development of a number of mobile apps, for example: SuckhoeVietnam, COVID-19, NCOVI, and VietnamHealth, supported the country’s prevention strategy as mobile phone penetration in the country is high [10]. However, as outlined earlier the accessibility of these apps was limited. Furthermore, although the government provided some accommodations, such as sign language on broadcasts, these were often poorly executed, with sign language presenters shown too small or signing too quickly, and failed to account for diverse needs, like different dialects of sign language [24]. Barriers such as technical jargon, low literacy, and limited access to information-communication technology further compounded these challenges, disproportionately affecting people with disabilities [24], who already face heightened risks of exclusion.

In addition to receiving information about COVID-19, people with disabilities reported difficulties adhering to COVID-19 preventive measures. Promisingly, people with disabilities reported being more likely to receive PPE. As most of the sample was recruited from Organizations of Persons with Disabilities, who often played a critical role in providing assistance to people with disabilities during the pandemic, these figures may be higher than for people not associated with these groups. Still, people with disabilities reported significant difficulties adhering to many COVID-19 preventive measures, including regular handwashing, hygiene protocols, social distancing, and wearing face masks. In other settings, common challenges included lack of information and understanding of protocols (particularly for people with intellectual disabilities), difficulties affording masks, soap and other resources, and their unsuitability for many people with disabilities (e.g., need for personal assistance complicates social distancing, people who lip read or use sign language face communication challenges when using masks) [28]. Vietnam’s stricter mask mandates, which included financial penalties, could have disproportionately affected people with disabilities [10], who were 1.6 times more likely (95% CI 1.2–2.1) to struggle with mask-wearing.

Vietnam’s vaccination efforts initially prioritized frontline workers and individuals at high risk of developing severe COVID-19 (although not explicitly people with disabilities), then expanded to the general population [9]. This priotization strategy may account for the lack of significant difference in vaccination rates between people with disabilities and people without disabilities (PR 1.0, 95% CI 0.9–1.1). However, people with disabilities encountered numerous obstacles during the vaccination process, including difficulties in obtaining information, securing appointments, and traveling to vaccination sites. The wide confidence interval for traveling to vaccination appointments may reflect differences in barriers experienced by impairment type (PR 24.5, 95% CI 9.2–65.7) [29]. Additionally, people with disabilities were also 2.4 times more likely (95% CI 1.7–3.5) to experience issues while waiting at the vaccination site and 11.9 times more likely (95% CI 4.8–29.5) to encounter problematic interactions with staff, highlighting the need for better accommodations for people with disabilities during the implementation of public health interventions. Despite these barriers, vaccination coverage among people with disabilities was similar to that of people without disabilities. This finding suggests that high coverage may have been achieved through the efforts of people with disabilities and their families to navigate inaccessible systems, rather than because vaccination processes were inherently inclusive. The experiences reported likely varied across impairment types. For example, people with mobility impairments may have faced greater physical barriers to travelling to vaccination sites, while people with hearing or visual impairments may have encountered greater challenges accessing information about vaccines or navigating digital systems. Individuals with intellectual or psychosocial impairments may also have experienced difficulties understanding complex public health guidance. Because mobility impairments were the most common impairment type among participants, some findings- particularly barriers related to travel - may reflect the experiences of this group more strongly than others.

Despite obstacles with adhering to preventive measures, people with disabilitieswere less likely to self-report COVID-19 symptoms, were equally likely to be tested for COVID-19, and less likely to be diagnosed compared to people without disabilities. This may suggest that people with disabilities in the study were less likely to acquire COVID-19, due to heavy shielding - as indicated by Ngoc et al. [24]. However, people with disabilities may also have been less able to leave home during the pandemic, which could have reduced infection risk independent of deliberate shielding behaviour. The greater use of rapid diagnostic tests amongst people with disabilities, which is less accurate than the reference standard of PCR testing [30], may also explain the lower proportion of participants with disabilities diagnosed with COVID-19 despite comparative levels of testing. Further exploration of this phenomenon would need to be undertaken to better understand the underlying reasons, and the effects on different impairment types. For instance, caregivers of people with communication difficulties may have difficulties identifying symptoms of COVID-19 and therefore this may be under-reported.

### COVID-19 effects on well-being

The COVID-19 pandemic had a detrimental impact on the well-being of the general population globally [31–36], and in Vietnam [37–41]. The fact that people with disabilities in Vietnam were 1.8 times more likely (95% CI 1.5–2.3) to report greater negative impacts on their well-being due to COVID-19, compared to those without disabilities, indicates that people with disabilities were particularly acutely affected. This has been seen in other studies [42, 43], where evidence indicates that those with chronic physical conditions, or neurodiversities have had a higher prevalence of COVID-19 related anxiety and depression symptoms – particularly amongst adolescents [42]. These findings underscore the need for tailored mental health interventions for people with disabilities during public health emergencies.

### Strengths and limitations

This study presents several strengths, including the collection of survey data during a time when conducting research was particularly challenging, and from areas most affected by COVID-19 in Vietnam [10]. This provided valuable insights into the experiences of people with disabilities during the pandemic. Additionally, Vietnam’s relatively good coverage of disability registration in urban areas enhances the representativeness of the findings.

However, the study also has limitations. Physical impairments were over-represented in the sample, potentially over-emphasising the influence of travelling to vaccination sites as a barrier compared with other impairment types. This may also influence the generalisability of findings to people with other impairment types. Phone interviews, necessitated by pandemic restrictions, resulted in lower response rates and may have excluded people with disabilities without access to mobile technology. This exclusion is likely tied to broader issues such as poverty, gender, and specific impairment types, which can limit access to technology. As noted by Banks et al. (2022) [44], adapting research methods during the pandemic posed significant challenges, particularly in reaching the most marginalized populations. Further research is also needed to explore the experiences of people with disabilities in rural areas.

## Conclusions

In conclusion, while Vietnam’s early response to the COVID-19 pandemic was largely successful, the experiences reported by people with disabilities reveal substantial gaps in accessibility and equity. Barriers to healthcare, information, and preventive measures point to the need for more targeted interventions to address these disparities. Although efforts such as the provision of PPE and hygiene products to people with disabilities indicate some level of support, persistent challenges in accessing services and adhering to preventive protocols demonstrate the necessity for further improvements.

The pandemic has underscored critical shortcomings in the enforcement of disability rights in Vietnam. Despite legal frameworks designed to protect the rights of people with disabilities, inconsistent implementation has limited their effectiveness, particularly during health emergencies [7, 45–47]. This study highlights the urgent need for more robust, inclusive policies that address equitable access to healthcare, education, and financial support for people with disabilities [6, 48]. While government efforts, such as vaccine distribution and healthcare services, were essential, they were often insufficient, reflecting broader systemic issues in achieving full inclusion [6]. Future emergency response planning must integrate the specific needs of this population to prevent the disproportionate impacts observed during the COVID-19 pandemic, thereby promoting greater equity and resilience in public health.

## Supplementary Information

Supplementary Material 1. Quantitative questionnaire used to collect quantitative data for this study.

## Data Availability

The dataset used is available from the corresponding author on reasonable request.

## Abbreviations

COVID-19: Corona Virus Disease 2019
OPD: Organisation of Persons with Disabilities
PCR: Polymerase Chain Reaction
PR: Prevalence Ratio
SARS: Severe Acute Respiratory Syndrome
