# Supplementary material for: Access to and self-reported health impacts of COVID-19 prevention measures on people with disabilities in Vietnam: Results from a cross-sectional study in three mega-cities
Source: BMC Public Health. 2026 Apr 14;26:1661. doi: 10.1186/s12889-026-27167-w (PMC13195997; doi:10.1186/s12889-026-27167-w)
Supplement: Supplementary file 1 — Supplementary Material 1. Quantitative questionnaire used to collect quantitative data for this study. [file 12889_2026_27167_MOESM1_ESM.docx]

**SECTION 1. Participant details**

1. Individual ID number:
2. Gender
   1. Male
   2. Female
3. Age (solar calendar):
4. Location (area):
   1. North
   2. South
   3. Central
5. Location:
   1. Rural
   2. Urban
6. Highest level of education?
   1. Primary school or lower
   2. Secondary school
   3. Highschool
   4. Undergraduate and higher
7. Is your household rated as a poor, or near poor? No; poor, near poor
8. Ethnicity
9. Religion
10. Marital status?
11. Single
12. Married
13. Separated/divorced/widow
14. Other/not response
15. Do you have social insurance?
    1. Yes
    2. No
16. Number of people in the household

**SECTION 2. Washington Group Short questions (ALL AGES)**

The next questions ask about difficulties you may have doing certain activities because of a HEALTH PROBLEM.

1. Do you have difficulty seeing, even if wearing glasses? [Read response categories]
   1. No – no difficulty
   2. Yes – some difficulty
   3. Yes – a lot of difficulty
   4. Yes – cannot do at all
2. Do you have difficulty hearing, even if using a hearing aid? [Read response categories]
   1. No – no difficulty
   2. Yes – some difficulty
   3. Yes – a lot of difficulty
   4. Yes – cannot do at all
3. Do you have difficulty walking or climbing steps? [Read response categories]
   1. No – no difficulty
   2. Yes – some difficulty
   3. Yes – a lot of difficulty
   4. Yes – cannot do at all
4. Do you have difficulty with self-care, such as washing all over or dressing? [Read response categories]
   1. No – no difficulty
   2. Yes – some difficulty
   3. Yes – a lot of difficulty
   4. Yes – cannot do at all
5. Using your usual language, do you have difficulty communicating, for example understanding or being understood? [Read response categories]
   1. No – no difficulty
   2. Yes – some difficulty
   3. Yes – a lot of difficulty
   4. Yes – cannot do at all
6. Do you have difficulty remembering or concentrating? [Read response categories]
   1. No – no difficulty
   2. Yes – some difficulty
   3. Yes – a lot of difficulty
   4. Yes – cannot do at all
7. Do you consider yourself to have a disability?
   1. Yes
   2. No
8. Do you have a disability card from the government?
   1. Yes
   2. No
9. [If 18=a]: What category of card?
   1. Extremely severe
   2. Severe
   3. Mild
   4. Don’t know
10. [If 18=a] What type of disability?
    1. Physical
    2. Hearing and speaking
    3. Visual
    4. Mental and psychiatric
    5. Intellectual
    6. Other, specify:

**Section 3. Employment (Age 15+: Q22-32; if <15, skip to Q31)**

1. Immediately before COVID-19 outbreak (around February 2020), were you working for pay or profit?
   1. Yes
   2. No
2. Have you worked for pay or profit since COVID-19 (since March 2020)?
   1. Yes
   2. No (skip to 27)
3. Which of the following best describes the type of work you do [main source of pay/profit]:
   1. Work for a company/non-family member
   2. Working for a family business/in self-employment
4. At any point since the start of COVID-19 (around March 2020) did you:
   1. Stop working Y N
   2. Work reduced hours Y N
   3. Worked more hours than usual for the same or less pay Y N
5. [If 24a, b, c= yes] How long did this go on for?

| 24.1. If stop working | 24.2. If work reduced hours | 24.3. Worked more hours than usual for the same or less pay |
| --- | --- | --- |
| - 1. <1 month   2. 1-3 months   3. 4-6 months   4. 7-11 months   5. 12+ months | 1. <1 month 2. 1-3 months 3. 4-6 months 4. 7-11 months 5. 12+ months | 1. <1 month 2. 1-3 months 3. 4-6 months 4. 7-11 months 5. 12+ months |

1. [If 25a, b = yes]: Did you receive any financial support from your employer because you stopped working/reduced your working hours?
   1. Yes
   2. No
2. [If 28=a & 25a, b=yes] Did you receive social health insurance payments when you stopped working, reduced your hours?
   1. Yes
   2. No
3. What best describes your earnings during the COVID-19 outbreak compared to the period before?
   1. Higher
   2. Same
   3. Lower
4. Did any other household member stop working during COVID-19?
   1. Yes
   2. No
5. [If 31=a] Did that household member receive:
   1. Social health insurance payments Y N
   2. Payments from employer Y N

**Section 4. Education (age 6-18)**

1. Dose your household have any child age 6-18 years old?
   1. Yes
   2. No [skip to next section]
2. Dose your child have any disability?
   1. Yes [repeat question 21, 22 for this child]
   2. No
3. Immediately before COVID-19 outbreak (around February 2020), were you in school?
   1. Yes
   2. No
4. Are you attending school now (including distance learning)?
   1. Yes
   2. No
5. [If 28=a and 29=b] Why are you no longer attending school? [Check all that apply]
   1. Graduated/finished as expected
   2. Working
   3. Married
   4. Can’t afford
   5. School closed
   6. School moved to distancing learning and can’t access
   7. Other, specify:
6. At any time during the COVID-19 outbreak did your school close completely, meaning no classes were offered either online or in-person?
   1. Yes
   2. No
7. [If 36=a] How long was your school closed completely (no classes at all, either in person or distance)?
   1. <1 week
   2. 1-2 weeks
   3. 3-4 weeks
   4. 1-2 months
   5. 2+ months
8. At any time during the COVID-19 outbreak did your school move to distance learning?
   1. Yes
   2. No
9. [If 38=a] How long did you do distance learning?
   1. <1 month
   2. 1-3 months
   3. 4-6 months
   4. 7-11 months
   5. 12+ months

| [If 38=a] To what extent, if any, did you face challenges with any of the following during distance learning? | |
| --- | --- |
| 1. Getting access to a computer and internet for your classes | 1. No difficulty 2. Some difficulty 3. A lot of difficulty |
| 1. Understanding lessons | 1. No difficulty 2. Some difficulty 3. A lot of difficulty |
| 1. [People with disabilities only]: Receiving education supports I needed due to my disability | 1. Not applicable 2. No difficulty 3. Some difficulty 4. A lot of difficulty |

1. Compared to before COVID-19, how well have you been able to keep in touch with your friends?
   1. A lot less
   2. A little less
   3. Same
   4. A little more
   5. A lot more

**Section 5. Disability-related health and social service needs (ALL AGES, People with disabilities only)**

|  | a. Before COVID-19 were you using…. | b. (If a=yes): Were you able to access [service] when needed during the COVID-19 outbreak? | c. (If a=no): Why not? |
| --- | --- | --- | --- |
| 1. Medications linked to your disability/health condition | Yes No | - Yes, same as before - Yes, with greater difficulty - Yes, with less difficulty - No - N/A | - No longer needed - Can’t afford - Provider unavailable due to Covid restrictions - I can no longer go to provider due to Covid restrictions - Other (specify): ....................   ....................................................  .................................................... |
| 1. Health services for your disability/ health condition | Yes No | - Yes, same as before - Yes, with greater difficulty - Yes, with less difficulty - No - N/A | - No longer needed - Can’t afford - Provider unavailable due to Covid restrictions - I can no longer go to provider due to Covid restrictions - Other (specify): ....................   ....................................................  .................................................... |
| 1. Assistive devices/products for disability/ health condition (e.g. glasses, wheelchair) | Yes No | - Yes, same as before - Yes, with greater difficulty - Yes, with less difficulty - No - N/A | - No longer need - Can’t afford - Provider unavailable due to Covid restrictions - I can no longer go to provider due to Covid restrictions - Other (specify): ....................   ....................................................  .................................................... |
| 1. Assistance/support for daily life activities, such as eating, dressing, bathing, using transportation? | Yes No | - Yes, same as before - Yes, with greater difficulty - Yes, with less difficulty - No - N/A | - No longer need - Can’t afford - Provider unavailable due to Covid restrictions - I can no longer go to provider due to Covid restrictions - Other (specify): ....................   ....................................................  .................................................... |

**Section 7. Self-reported impact of COVID-19 and access to services**

| What has been the impact of the COVID-19 outbreak and related restrictions on….? | Answer options:   1. Large negative 2. Somewhat negative 3. No change 4. Somewhat positive 5. Large positive 6. N/A |
| --- | --- |
| 1. Your household finances |  |
| 1. Your ability to get the food you need |  |
| 1. Your access to general medication and healthcare |  |
| 1. Your well-being (e.g. boredom, loneliness, anxiety, stress) |  |
| 1. Your social life |  |

| To what extent, if any, have you faced difficulty with the following during the COVID-19 prevention measures? | Answer options:   - 1. Not at all   2. Some difficulty   3. A lot of difficulty |
| --- | --- |
| 1. Wearing a mask/face covering |  |
| 1. Regular handwashing and other hygiene procedures |  |
| 1. Social distancing |  |

| Since the COVID-19 outbreak, have you received any of the following from the government or an organisation? |  |
| --- | --- |
| 1. Food deliveries, food assistance | Yes No |
| 1. Financial assistance (e.g. cash transfer, package for vulnerable groups) | Yes No |
| 1. Personal protection, hygiene products (e.g. soap, face masks, sanitiser) | Yes No |
| 1. Distance learning products (e.g.Laptop, tablet, mobile phone) | Yes No |
| 1. Social distancing support (e.g. free internet, reduce price of electricity) | Yes No |

**Section 8. Vaccines**

1. Have you been vaccinated for COVID-19?
   1. Yes, I’m fully vaccinated
   2. Yes, but I’m not fully vaccinated
   3. No
   4. Don’t know
2. [If 61=c] Do you want to receive the COVID-19 vaccine?
   1. Yes
   2. No
   3. Don’t know
3. [If 61=c] People may not have received the vaccination for a number of reasons. What are the reasons why you have not been vaccinated? [Check all that apply]
   1. I don’t think I’m eligible
   2. I have a health condition that doesn’t allow me to get vaccinated at the moment
   3. Don’t know where to go
   4. Worried about costs
   5. Too far
   6. I am concerned about the vaccine’s safety
   7. I don’t need it
   8. Other (specify):

| [If 61=a, b] To what extent, if at all, were the following a challenge when getting your vaccine? | |
| --- | --- |
| 1. Getting information about the vaccine | No difficulty  Some difficulty  A lot of difficulty |
| 1. Getting an appointment | No difficulty  Some difficulty  A lot of difficulty |
| 1. Traveling to the appointment site | No difficulty  Some difficulty  A lot of difficulty |
| 1. Waiting at the vaccine site to be seen | No difficulty  Some difficulty  A lot of difficulty |
| 1. Interactions with staff at the vaccine site | No difficulty  Some difficulty  A lot of difficulty |
| 1. Overall, how satisfied were you with the process for getting vaccine at your area? | Very unsatisfied  Somewhat unsatisfied  Neither satisfied or dissatisfied  Somewhat satisfied  Very satisfied |

**Section 9. COVID-19**

1. Have you ever experienced symptoms of COVID-19 such as fever, cough or shortness of breath since COVID-19?
   1. Yes
   2. No
2. (If 70=a): Have you ever been tested for COVID-19?
   1. Yes, I did quick test
   2. Yes, I did PCR test
   3. No
3. (If 71=a) Were you diagnosed with Covid-19?
   1. Yes
   2. No
4. (If 72=a) What happened after you were diagnosed?
   1. Home isolation with check in from community health worker
   2. Be delivered to government' isolation camp
   3. Be delivered to hospital
   4. Other (specify):

**Section 10. YOUR HEALTH TODAY.**

Please select the ONE box that best describes your health TODAY

1. MOBILITY
2. I have no problems in walking about □
3. I have slight problems in walking about □
4. I have moderate problems in walking about □
5. I have severe problems in walking about □
6. I am unable to walk about □
7. SELF-CARE
8. I have no problems washing or dressing myself □
9. I have slight problems washing or dressing myself □
10. I have moderate problems washing or dressing myself □
11. I have severe problems washing or dressing myself □
12. I am unable to washing or dressing myself □
13. USUAL ACTIVITIES (e.g. work, study, housework, family or leisure activities)
14. I have no problems doing my usual activities □
15. I have slight problems doing my usual activities □
16. I have moderate problems doing my usual activities □
17. I have severe problems doing my usual activities □
18. I am unable to do my usual activities □
19. PAIN/DISCOMFORT
20. I have no pain or discomfort □
21. I have slight pain or discomfort □
22. I have moderate pain or discomfort □
23. I have severe pain or discomfort □
24. I have extreme pain or discomfort □
25. ANXIETY/ DEPRESSION
26. I have not anxious or depressed □
27. I am slight anxious or depressed □
28. I am moderate anxious or depressed □
29. I am severe anxious or depressed □
30. I am extreme anxious or depressed □
31. VAS

**
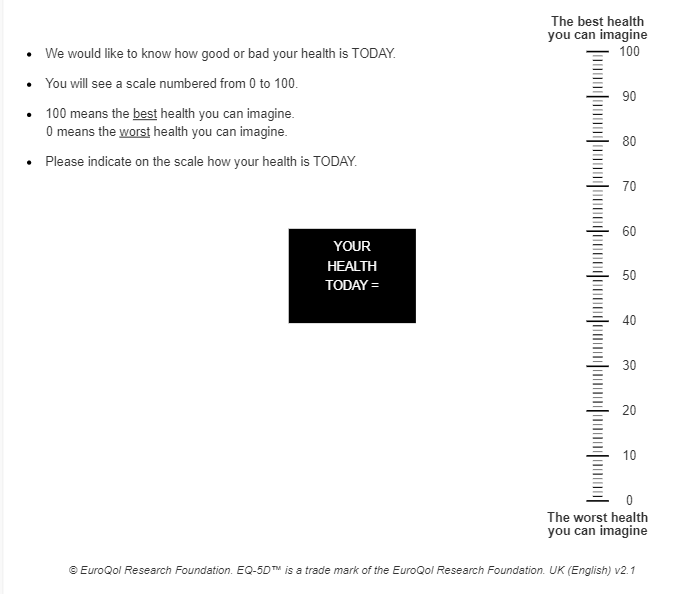
**

**Section 11. Depression, Anxiety and Stress Scale - 21 Items (DASS-21)**

Please read each statement and circle a number 0, 1, 2 or 3 which indicates how much the statement applied to you over the past week. There are no right or wrong answers. The rating scale is as follows:

0: Did not apply to me at all

1: Applied to me to some degree, or some of the time

2: Applied to me to a considerable degree or a good part of time

3: Applied to me very much or most of the time

| No. | Statements | Scores | | | |
| --- | --- | --- | --- | --- | --- |
|  | I found it hard to wind down | 0 | 1 | 2 | 3 |
|  | I was aware of dryness of my mouth | 0 | 1 | 2 | 3 |
|  | I couldn’t seem to experience any positive feeling at all | 0 | 1 | 2 | 3 |
|  | I experienced breathing difficulty (e.g. excessively rapid breathing, breathlessness in the absence of physical exertion) | 0 | 1 | 2 | 3 |
|  | I found it difficult to work up the initiative to do things I tended to over-react to situations | 0 | 1 | 2 | 3 |
|  | I experienced trembling (e.g. in the hands) | 0 | 1 | 2 | 3 |
|  | I felt that I was using a lot of nervous energy | 0 | 1 | 2 | 3 |
|  | I was worried about situations in which I might panic and make a fool of myself | 0 | 1 | 2 | 3 |
|  | I felt that I had nothing to look forward to I found myself getting agitated | 0 | 1 | 2 | 3 |
|  | I found it difficult to relax | 0 | 1 | 2 | 3 |
|  | I felt down-hearted and blue | 0 | 1 | 2 | 3 |
|  | I was intolerant of anything that kept me from getting on with what I was doing | 0 | 1 | 2 | 3 |
|  | I felt I was close to panic | 0 | 1 | 2 | 3 |
|  | I was unable to become enthusiastic about anything | 0 | 1 | 2 | 3 |
|  | I felt I wasn’t worth much as a person | 0 | 1 | 2 | 3 |
|  | I felt that I was rather touchy | 0 | 1 | 2 | 3 |
|  | I was aware of the action of my heart in the absence of physical exertion (e.g. sense of heart rate increase, heart missing a beat) | 0 | 1 | 2 | 3 |
|  | I felt scared without any good reason I felt that life was meaningless | 0 | 1 | 2 | 3 |
